# M. Velpeau’s Report on Coal-tar as a Disinfectant

**Published:** 1860-07

**Authors:** 


### M. VELPEAU'S REPORT ON COAL-TAR AS A DISINFECTANT.

The Academy of Sciences having appointed a committee composed of MM. Cheveul, Cloquet, and Velpeau, to consider the claims of MM. Corne and Demeaux's preparation of coal-tar and plaster as a disinfectant, an extensive series of compar ative trials between it and other substances was instituted at La Charite, and M. Velpeau has recently presented to the Academy an elaborate report on the results. The general conclusions are—1. That coal-tar mixed with plaster, disinfects putrefying organic matter. 2. In its therapeutical applications it has only realized a portion of its promises. As a disinfectant in the autopsy-rooms, (upon this point even in the hottest weather, M. Velpeau speaks in the strongest terms,) in the beds of the dirty patients, and wherever there are infected matters, its properties are indubitable. The same may be said of putrid and gangrenous discharges, fetid suppurations, sanious wounds, ichorous caverns, and hospital gangrene; but with respect to open wounds and ordinary ulcers, other topical preparations are preferable. 3. Employed with charpie, linen, ointments, cerates, or taken internally, it has furnished no useful result. 4. As an absorbent, especially used as a cataplasm, it acts very indifferently. It must be remembered that abnormal liquids, especially pus, are very different compounds to water; and plaster, which exerts great absorbing power on the latter, may imbibe but very little pus. Still, whether in powder or cataplasms, the compound may render some service, even as an absorbent, in fetid or putrid suppurations. To secure the good effects of the coal-tar and plaster, certain percautions must be taken, the neglect of which may probably explain the entire inefficacy this mixture has exhibited in the hands of some experimenters. It is fine modeling plaster, and not common plaster, which should be employed; and the coal-tar, or carburetted oils, in the proportion of from two to four per cent, triturated with it, should impart to it a grayish color, while leaving its dry and pulverulent quality. Anatomical parts, to be disinfected, should be rolled in this powder, so as to bring it in contact with all parts at their surfaces. Gangrenous or putrid sores, must be covered by thick layers, laid on in handfuls several times a day; and, in the case of blood, pus, dejections, etc., enough must be put on to form a kind of paste, replacing the layer of powder by a new one, when it no longer absorbs. Mixed with white oil into a thick state, it may be applied as a cataplasm.—*Med. Times.*

